# Clinical feasibility and validation of 3D principal strain analysis from cine MRI: comparison to 2D strain by MRI and 3D speckle tracking echocardiography

**DOI:** 10.1007/s10554-017-1199-7

**Published:** 2017-07-06

**Authors:** Alessandro Satriano, Bobak Heydari, Mariam Narous, Derek V. Exner, Yoko Mikami, Monica M. Attwood, John V. Tyberg, Carmen P. Lydell, Andrew G. Howarth, Nowell M. Fine, James A. White

**Affiliations:** 1 Stephenson Cardiac Imaging Centre, University of Calgary, Suite 0700 Foothills Medical Centre – 1403 29th St NW, Calgary, AB T2N 2T9 Canada; 2 Division of Cardiology, Department of Cardiac Sciences, Libin Cardiovascular Institute of Alberta, University of Calgary, Calgary, AB Canada; 3 Department of Diagnostic Imaging, University of Calgary, Calgary, AB Canada

**Keywords:** Cardiovascular MRI, 3-Dimensional, Strain, Principal strain, Feature tracking

## Abstract

Two-dimensional (2D) strain analysis is constrained by geometry-dependent reference directions of deformation (i.e. radial, circumferential, and longitudinal) following the assumption of cylindrical chamber architecture. Three-dimensional (3D) principal strain analysis may overcome such limitations by referencing intrinsic (i.e. principal) directions of deformation. This study aimed to demonstrate clinical feasibility of 3D principal strain analysis from routine 2D cine MRI with validation to strain from 2D tagged cine analysis and 3D speckle tracking echocardiography. Thirty-one patients undergoing cardiac MRI were studied. 3D strain was measured from routine, multi-planar 2D cine SSFP images using custom software designed to apply 4D deformation fields to 3D cardiac models to derive principal strain. Comparisons of strain estimates versus those by 2D tagged cine, 2D non-tagged cine (feature tracking), and 3D speckle tracking echocardiography (STE) were performed. Mean age was 51 ± 14 (36% female). Mean LV ejection fraction was 66 ± 10% (range 37–80%). 3D principal strain analysis was feasible in all subjects and showed high inter- and intra-observer reproducibility (ICC range 0.83–0.97 and 0.83–0.98, respectively—p < 0.001 for all directions). Strong correlations of minimum and maximum principal strain were respectively observed versus the following: 3D STE estimates of longitudinal (r = 0.81 and r = −0.64), circumferential (r = 0.76 and r = −0.58) and radial (r = −0.80 and r = 0.63) strain (p < 0.001 for all); 2D tagged cine estimates of longitudinal (r = 0.81 and r = −0.81), circumferential (r = 0.87 and r = −0.85), and radial (r = −0.76 and r = 0.81) strain (p < 0.0001 for all); and 2D cine (feature tracking) estimates of longitudinal (r = 0.85 and −0.83), circumferential (r = 0.88 and r = −0.87), and radial strain (r = −0.79 and r = 0.84, p < 0.0001 for all). 3D principal strain analysis is feasible using routine, multi-planar 2D cine MRI and shows high reproducibility with strong correlations to 2D conventional strain analysis and 3D STE-based analysis. Given its independence from geometry-related directions of deformation this technique may offer unique benefit for the detection and prognostication of myocardial disease, and warrants expanded investigation.

**Electronic supplementary material:**

The online version of this article (doi:10.1007/s10554-017-1199-7) contains supplementary material, which is available to authorized users.

## Introduction

Two-dimensional (2D) strain analysis has emerged as a reproducible method for identifying altered ventricular function in patients with cardiovascular disease [1, 2]. However, left ventricular (LV) deformation is a 3-dimensional (3D) process representing composite contributions from counter-directional, helically arranged fibers shortening and thickening throughout the cardiac cycle [3]. Diseases of the myocardium uniquely and regionally influence these fibers and therefore present unique opportunities to exploit fiber-sensitive measures of myocardial deformation to discriminate disease phenotype. 2D strain represents net deformation in pre-defined geometry-related directions, these assuming cylindrical chamber architecture. Accordingly, such pre-defined directions of deformation may not reliably describe deformation in the dominant direction of tissue deformation, established by engaged myocardial fibers.

While 3D image acquisition and reconstruction techniques for quantifying tissue deformation have expanded significantly for cardiovascular magnetic resonance (CMR) [4], echocardiography [5, 6], and gated computerized tomography angiography (CTA) [7, 8], resultant analyses have historically remained constrained to geometry-dependent directions of deformation (i.e. longitudinal, circumferential and radial directions). In this study, we explore the feasibility of 3D strain analysis from routine cine CMR images for the estimation of principal strain, a geometry-independent measure established from the dominant direction of local tissue deformation (12), thus providing a ubiquitous description of tissue contraction relevant to any chamber architecture.

## Methods

### Study population

Thirty-one consecutive patients ≥18 years of age clinically referred for CMR imaging at the Stephenson Cardiac Imaging Centre were recruited. Patients with any clinical indication other than complex congenital heart disease (including known or suspected CAD) were considered eligible. All patients over 18 years of age clinically referred for CMR imaging were considered eligible with exception of patients with complex congenital heart disease or atrial fibrillation. All patients were asked to undergo additional transthoracic echocardiography imaging with 3D STE within 4 weeks of CMR. Additionally, patients with standard contraindications to CMR imaging were not studied.

This study was approved by the University of Calgary Research Ethics Board and all patients provided informed consent to participate.

### Cardiac magnetic resonance imaging protocol

CMR imaging was performed using a clinical 1.5-T MRI system (Avanto^®^, Siemens Healthcare, Erlangen Germany) with a 32-channel cardiac coil and retrospective ECG gating. The CMR imaging protocol included standard, end-expiratory cine imaging in sequential short axis (SAX) planes (from above the mitral valve annulus to beyond the LV apex) and long axis (LAX) planes (in 2, 3 and 4-chambers views) using a steady-state free-precession (SSFP) pulse sequence. Typical imaging parameters were: slice thickness 8 mm, gap 2 mm, TE 1.5 ms, flip angle 50 degrees, matrix 256 × 205, in-plane spatial resolution 1.5 × 1.5 mm, temporal resolution 30–45 ms, acceleration factor (iPAT) of 2, 30 phases per cardiac cycle. For validation purposes 2D tagged cine imaging spatially matched to SAX and LAX cine imaging was incrementally performed for 15 patients. Typical imaging parameters for tagged MR imaging were as follows: Echo Time (TE) 2.55 ms, Repetition Time (TR) 59 ms, 10° flip angle, slice thickness 10 mm, gap 0 mm, 224 × 144 matrix, iPat 2, 30 phases per cardiac cycle.

LV volumes and mass were determined from cine images using commercial software (cvi^42^, Circle Cardiovascular Imaging Inc, Calgary Canada) with manual tracing of endocardial and epicardial borders at end–diastole and end-systole. LV volumes and mass were indexed to body surface area (BSA). The papillary muscles were included as part of the LV mass.

### Cardiac magnetic resonance imaging: 3D strain analysis

#### LV mesh modeling

**Fig. 1:**
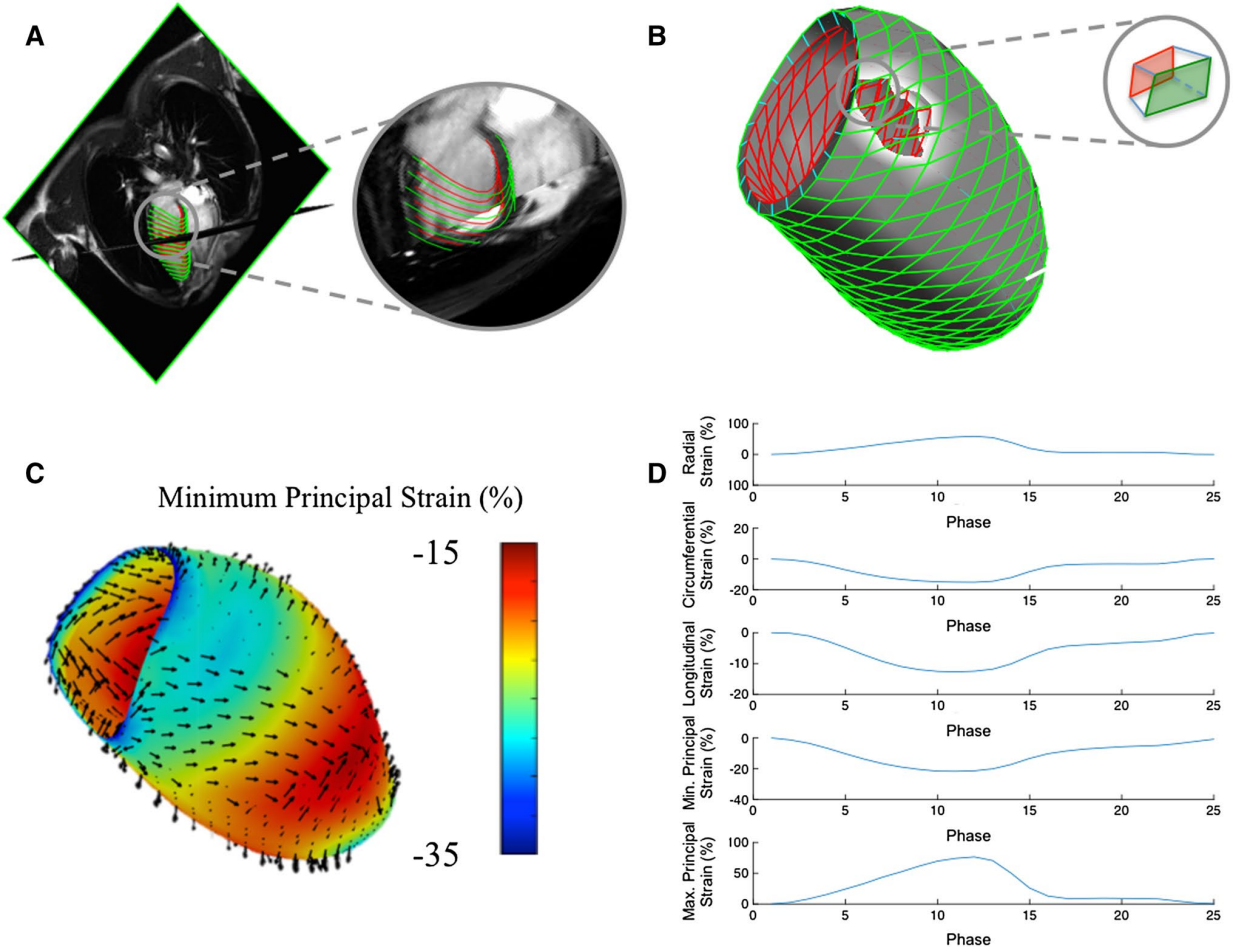
Software workflow for the calculation of 3D LV strain from 2D CMR cine imaging. Contours are applied to long axis cine images to construct endocardial and epicardial surface mesh models (**a**). A transmural hexahedral mesh is constructed (**b**), enabling the calculation of endocardial, epicardial and transmural strain from respective quadrangular components of each hexahedron.** c** Illustrates a 3D LV strain *color* map of endocardial principal strain and directions thereof at peak systole.** d** Provides global transmural strain curves for principal strain and conventional geometry-dependent directions

Locally developed Matlab-based software was used to perform 3D LV strain analysis (version R2014b, The MathWorks, Natick Massachusetts), an expansion of previously described work [9]. All routinely acquired SAX and LAX images were imported in standard, uncompressed DICOM format [10] and automatically co-registered according to their Cartesian coordinates. Manual correction of persistent misalignments (related to marked breath hold variations) was performed by rigid transformation (Fig. 1). Epicardial and endocardial contours were then traced for a single end-diastolic frame for each of the 3 LAX images to generate a 2-layer mesh model of the LV by means of a spline-based approach, as originally described by De Boor [11]. This mesh model was subsequently subjected to a mesh-smoothing algorithm [12] and corresponding points (nodes) from respective endocardial and epicardial surfaces coupled to obtain a hexahedral (transmural) 3D mesh model of the LV [13]. Typically, each LV mesh model was composed of approximately 700 hexahedral elements available for the calculation of single surface (endocardial, epicardial) or dual surface (transmural) strain [14], as further described in Appendix 1. An example of a 3D LV mesh model from an individual with no cardiovascular findings is shown in Fig. 1.

#### Motion and strain evaluation

**Fig. 2:**
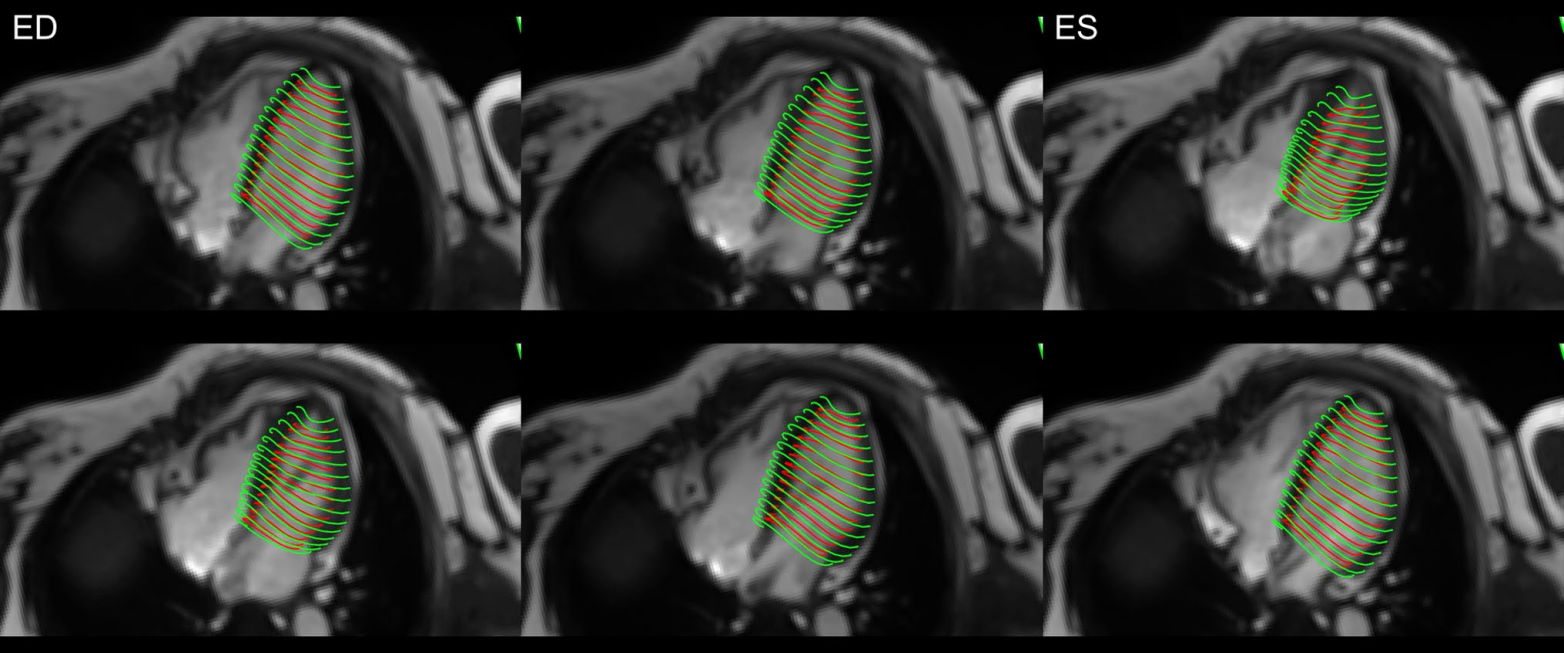
Screenshots taken at equidistant phases throughout the cardiac cycle to reflect relative changes in both subendocardial and subepicardial LV tracked borders, as referenced by 4-chamber cine images. ED and ES indicate which phases correspond to end diastole and end systole, respectively

All co-registered cine images contributed to the development of a 4D displacement field [15–17], achieved through application of previously validated feature-tracking based algorithms [18, 19] to each and every voxel of the cine images. Computed velocities were used to derive virtual displacements for a dense, isotropic field over the imaging volume and used to deform the mesh models obtained from the original image dataset throughout the cardiac cycle (Supplemental On-line Video). In the current application, each node of the hexahedral LV elements (8 per hexahedron) were assigned a 3-dimensional weighted velocity, considering both their geographic vicinity and orientation relative to computed voxel displacements. The velocity between time-steps *n* and *n* + 1 was expressed in terms of spatial units (mm) per time step, thus allowing for the conversion of velocity into an incremental displacement in the material point between the two consecutive time steps. Deformations for each hexahedral element were then calculated using a Lagrangian strain definition [14] at each cardiac phase (with respect to the end-diastolic phase). Principal strain (PS) was calculated for each element, both for each individual surface (endocardial and epicardial) and for their transmural interaction. For comparison, longitudinal and circumferential strain were also calculated for nodal elements of the endocardial and epicardial surfaces with radial strain calculated from their transmural interaction. An example of the independent tracking of the endocardial and epicardial surfaces is shown in Fig. 2.

**Fig. 3:**
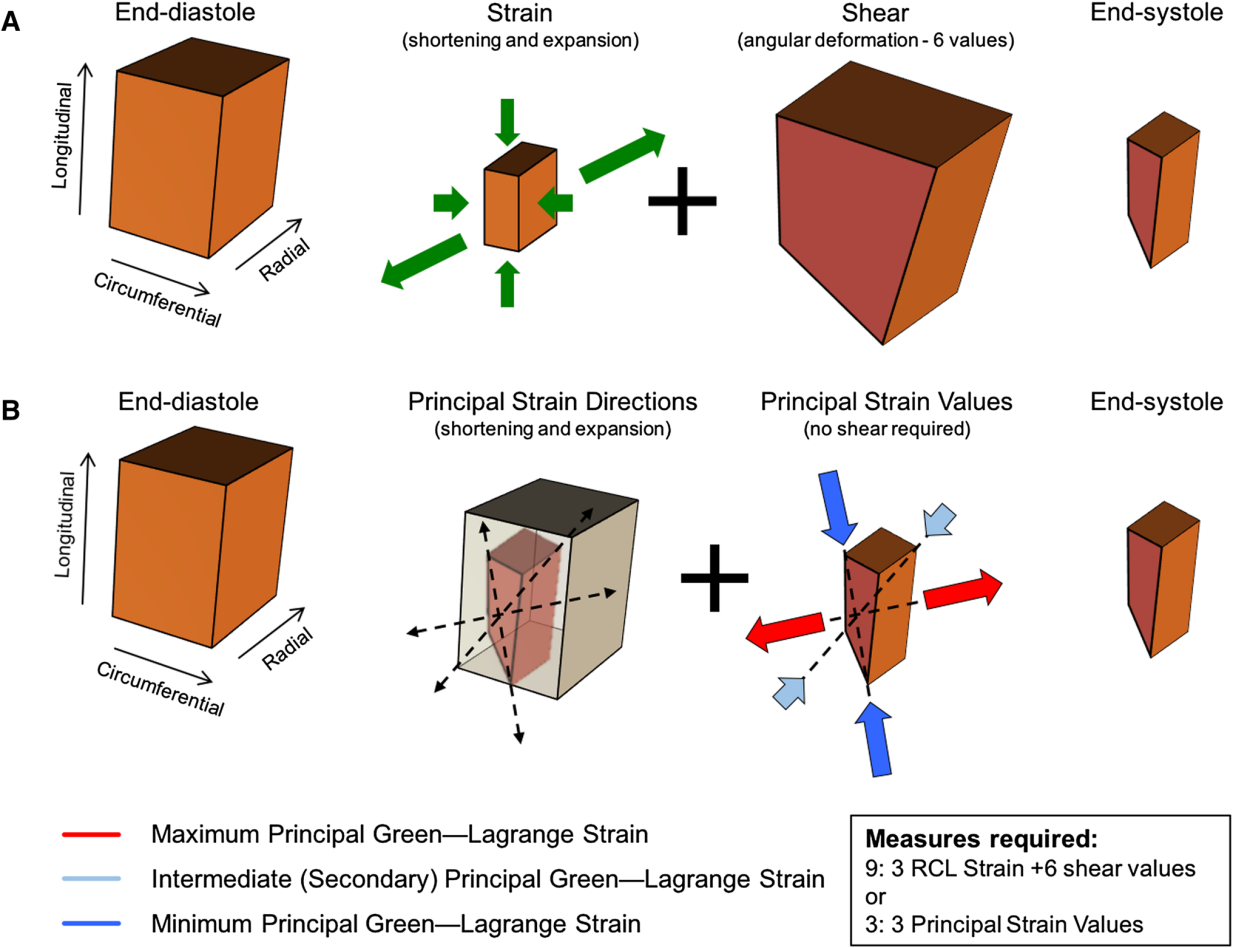
Pictorial summary of the definition of principal strain. **a** The deformation of a tissue element from its initial (end-diastolic) to a final (end-systolic) configuration is constituted of longitudinal and circumferential shortening, plus radial expansion (thickening) and 6 angular deformations (shear deformation). When using only three geometry-dependent directions (radial, circumferential and longitudinal), strain obtained in those directions cannot account for shear and, therefore, does not offer a complete description of the strain undergone by the element. **b** However, the same deformation can be described without shear in terms of principal strain along 3 principal directions, these established through a comparison of the initial and final configurations of the tissue element

As illustrated in Fig. 3, PS describes 3 orthogonal (principal) directions that completely portray the deformation experienced by an element without need for shear components, or a need to constrain analyses to conventional geometry-dependent directions (i.e.: radial, circumferential and longitudinal). While three PS values can be derived (maximum or “first”, intermediate or “second”, and minimum or “third” PS), the majority of lengthening (i.e. thickening) occurs in the maximum PS direction (typically a positive value), while the majority of shortening occurs in the minimum PS direction (typically a negative value) [3]. Additionally, because of tissue incompressibility, the intermediate principal strain is fully dependent on maximum and minimum PS. For these reason, intermediate PS may be reasonably excluded from the analysis output. Overall, this allows PS to provide a ubiquitous, simplified yet more comprehensive two-component strain assessment for all cardiovascular chambers.

### 3D echocardiography and strain analysis

Transthoracic echocardiography was performed in all subjects using the same commercially available ultrasound system (Vivid E9, GE Healthcare, Milwaukee WI). Imaging was performed according to American Society of Echocardiography guidelines using a standard protocol [20]. Incrementally 3D images were acquired in full volume-mode with the focus on the LV chamber from a single cardiac cycle during coordinated breath holds. Frame rate was optimized to >20 frames/s by focusing imaging sector size. Volumetric strain analysis of the 3D images was performed blinded to all clinical and CMR data using a commercially available software system (4D LV-Analysis version 3.0; TomTec Imaging Systems, Unterschleissheim, Germany). Following manual assignment of endocardial contours in three standard LAX views, the software uses a speckle tracking algorithm to generate a 3D LV endocardial surface mesh model throughout the cardiac cycle [3]. Endocardial longitudinal, circumferential and minimum PS are provided. The software also estimates an epicardial surface according to a mathematical relationship between the length of the initial contour in the sub-endocardial zone and the local myocardial wall thickness, thus allowing for an estimate of radial strain. Given the lower temporal resolution of 3D echo-based versus CMR-based strain analysis, each data point from echo-based strain curves was matched to the closest temporal data point of corresponding CMR-based strain curves for comparison purposes. This approach was chosen in order to avoid possible artifacts related to the resampling of data.

### 2D MRI strain analysis

2D MRI-based strain analysis was performed using both tagged cine images (tag deformation analysis) and non-tagged cine images (2D feature-tracking analysis) using commercially available software (Medviso AB, Lund, Sweden). This was performed using SAX views to estimate radial and circumferential strain, and LAX views to estimate longitudinal strain. All strain measurements were provided as global values for correlation to the tested 3D CMR strain analysis technique.

### Intra- and inter-observer reproducibility

CMR based 3D LV strain measurements were tested for intra-observer reproducibility by having one observer perform all CMR strain analyses on 10 randomly selected patients and then blindly repeating the analysis on a separate occasion. Inter-observer reproducibility was evaluated by a second observer, blinded to clinical and experimental data, performing CMR 3D LV strain analyses on the same 10 patients.

### Statistical analysis

Categorical variables are presented as percentages, whereas continuous variables are expressed as mean ± standard deviation or as median values with interquartile range depending on normality of the variable. Categorical variables were compared using the Fisher’s exact test, while comparisons for continuous data was performed using 2-sample independent t-test or Wilcoxon rank-sum test, where appropriate. To assess agreement between 3D LV conventional strain and PS analysis calculated over the full cardiac cycle using CMR and STE techniques, we used Pearson rank-order correlation coefficients. Linear regression modeling was independently performed for 2D CMR and 3D STE-based strain and 3D CMR-based strain calculations, with scatter plots presented for visual assessment.

Because the available 3D echocardiography based strain analysis software provided only endocardial values for longitudinal, circumferential and minimum PS, the comparison of these values to CMR 3D LV strain were limited to this surface. Both intra- and inter-observer agreement were analyzed by both ICC (with 95% CI) and Bland–Altman analysis. All statistical analysis was performed using commercially available software (SAS version 9.4, SAS Institute, Cary NC). A two-sided p-value of less than 0.05 was considered statistically significant.

## Results

### Patient population

Baseline clinical and CMR characteristics for the study population are presented in Table 1. The mean age was 51 ± 14 years and 36% were female. All patients were in sinus rhythm at the time of imaging. The mean LV ejection fraction (EF) was 66 ± 10% and ranged from 37 to 80%. The mean time interval between CMR and echocardiography examinations was 26 ± 15 days.

**Table 1:** Baseline clinical and CMR characteristics of the study population

Parameter	Study population (N = 31)
Clinical characteristics	
Age (years)	51 (±14)
Gender, male	20 (64%)
Body surface area (m^2^)	1.9 (±0.3)
Body mass index (kg/m^2^)	26.3 (±4.8)
Coronary artery disease	6 (19%)
Stroke	2 (6%)
Hypertension	8 (26%)
Diabetes mellitus	3 (10%)
Hyperlipidemia	7 (23%)
Medications	
Aspirin	11 (35%)
Beta-blocker	10 (32%)
ACE-inhibitor/ARB	8 (26%)
Cholesterol lowering agent	6 (19%)
Oral anticoagulant	5 (16%)
Nitrates	2 (6%)
CMR characteristics	
LV ejection fraction (%)	66 (±10)
LV end-diastolic volume (mL)	137 (±48)
LV end-systolic volume (mL)	50 (±32)
LV mass index (g/m^2^)	59 (±13)

The indications for clinical CMR referral among the study cohort were: known or suspected cardiomyopathy (n = 21), vascular assessment (pulmonary or aortic) (N = 5), first degree family screening (n = 3), valvular heart disease (N = 2). Final diagnosis was ischemic cardiomyopathy in 4 patients, dilated cardiomyopathy in 3, hypertrophic cardiomyopathy in 4, aortic or pulmonary vascular abnormality in 5, bicuspid aortic valve in 2 patients and 13 patients were found to be normal.

**Fig. 4:**
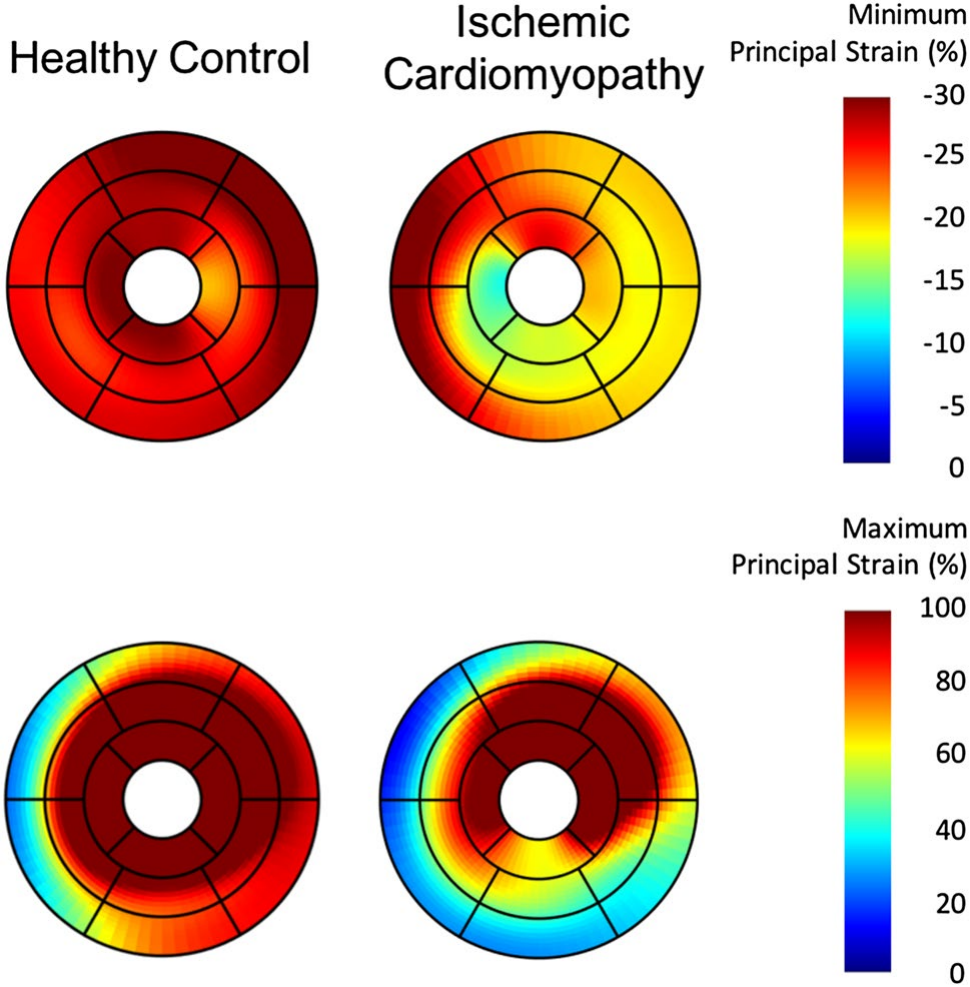
Case examples of principal strain analysis in a patient with no cardiovascular findings, and in a patient with ischemic cardiomyopathy. The *upper* pane reports minimum principal strain. The *lower* pane reports maximum principal strain. The latter patient demonstrates marked reductions in principal strain within the inferolateral wall, consistent with the presence of a transmural myocardial infarction (confirmed on LGE imaging)

Three-dimensional LV strain analysis from routine multi-planar cine imaging was feasible for all patients. Image quality was acceptable in all cases. No study was excluded due to poor image quality. The mean analysis time from initial image import (to off-line software) through to data presentation and dynamic 3D model display was 15 ± 2 min. Computational analysis time of all 3D strain values was 44 ± 12 s. Case examples of 3D-CMR PS analysis performed in subjects with varying systolic function are shown in Fig. 4. Strain values as computed by 3D CMR and 3D STE are reported in Table 2.

**Table 2:** Left ventricular 3-dimensional conventional and principal strain values calculated using cardiovascular magnetic resonance imaging (CMR) and echocardiography based approaches

Parameter	CMR	STE
Conventional strain		
Longitudinal		
Endocardial	−15.1 (±2.8)	−16.2 (±3.5)
Epicardial	−9.6 (±1.8)	
Transmural	−12.3 (±2.2)	
Circumferential		
Endocardial	−18.3 (±3.3)	−20.9 (±5.9)
Epicardial	−9.8 (±2.1)	
Transmural	−13.7 (±2.5)	
Radial		
Transmural	43.7 (±13.3)	66.8 (±24.4)
Principal strain		
Minimum		
Endocardial	−23.0 (±3.6)	−31.6 (±6.0)
Epicardial	−17.0 (±2.9)	
Transmural	−19.9 (±3.1)	
Maximum		
Transmura	62.4 (±18.3)	

Peak systolic strain values (%) are presented as the mean (±standard deviation). Endocardial, epicardial and transmural myocardial strain values are presented for CMR based strain calculations, whereas only endocardial surface and radial strain values were available for echocardiography. *CMR* cardiovascular magnetic resonance imaging, *STE* speckle tracking echocardiography

### 3D CMR principal strain versus 3D geometry-dependent strain

Comparisons of 3D CMR PS versus 3D STE-based strain in geometry-dependent directions calculated from both CMR (internal validation) and STE (external validation) are presented in Table 3. Strong correlations were identified for both sets of analyses. Of particular note, external validation of 3D CMR-PS (maximum and minimum) versus geometry-dependent 3D-STE based measures of strain (Table 3) was robust with maximum PS (transmural) strongly correlated to radial (transmural), circumferential (endocardial) and longitudinal (endocardial) strain. The respective correlation coefficients were: 0.63 (p < 0.0001), −0.58 (p < 0.0001), and −0.64 (p < 0.0001). Minimum PS (endocardial) was similarly correlated to the same strain measures with correlation coefficients of −0.80 (p < 0.0001), 0.76 (p < 0.0001), and 0.81 (p < 0.0001), respectively.

**Table 3:** Correlation coefficients for 3D CMR measures of principal strain and STE-based strain measure in geometry-dependent directions

Principal strain (3D CMR)	Strain measures in geometry-dependent directions	r-value (vs. 3D CMR geometry-dependent strain)	r-value (vs. 3D STE geometry-dependent strain)
Maximum principal strain			
	Radial strain	0.75 (p < 0.0001)	0.63 (p < 0.0001)
	Circumferential strain	−0.78 (p < 0.0001)	−0.58 (p < 0.0001)
	Longitudinal strain	−0.80 (p < 0.0001)	−0.64 (p < 0.0001)
Minimum principal strain			
	Radial strain	−0.92 (p < 0.0001)	−0.80 (p < 0.0001)
	Circumferential strain	0.98 (p < 0.0001)	0.76 (p < 0.0001)
	Longitudinal strain	0.98 (p < 0.0001)	0.81 (p < 0.0001)

Data are expressed as Pearson correlation coefficients (r) (p-value)

### 3D CMR principal strain versus 3D STE minimum principal strain

**Fig. 5:**
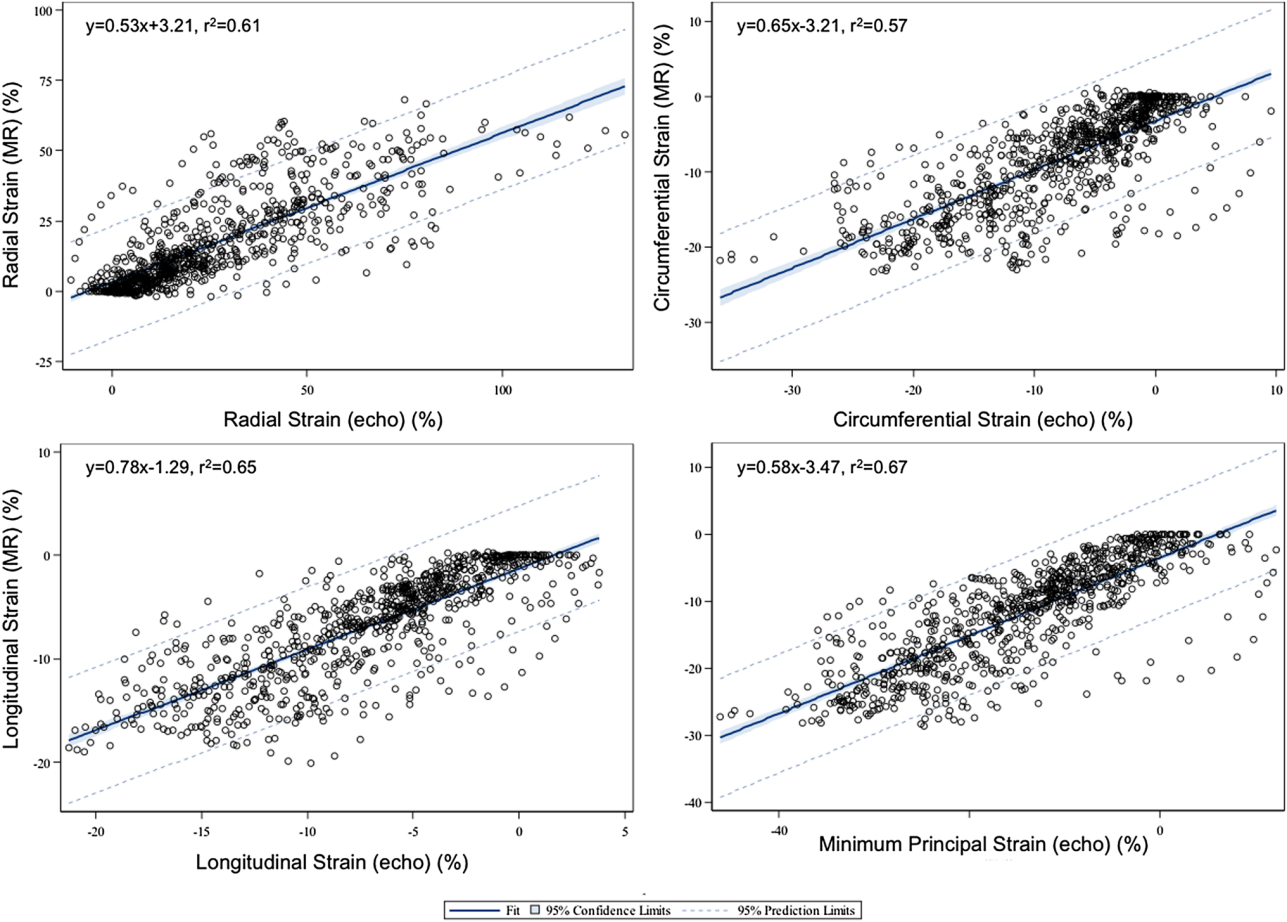
Scatter plots with line of best fit (linear regression) for *left* ventricular 3D minimum principal strain and conventional longitudinal, circumferential and radial strain from cardiovascular magnetic resonance imaging versus those available from 3D speckle-tracking echocardiography. The *dotted lines* are the 95% confidence interval prediction limits

A measure of minimum PS at the endocardial layer was available from the 3D STE analysis software used in this study. Accordingly, a cross-platform comparison of this measure was feasible. Regression analysis showed a high Pearson correlation rank of r = 0.82 (p < 0.001) (Fig. 5) with ICC of 0.74 (95% CI 0.71–0.77), consistent with strong agreement. As maximum PS requires tracking of both the endocardial and epicardial surfaces, this was not available from 3D-STE.

### 3D CMR geometry-dependent strain versus 3D STE geometry-dependent strain

To compare tissue deformation analysis between modalities using like measures, strain measures calculated in conventional geometry-dependent directions using the 3D CMR model were compared to those available from 3D-STE. These showed strong correlation for each of the longitudinal, circumferential and radial directions. Corresponding Pearson correlation coefficients for longitudinal, circumferential and radial strain were as follows: r = 0.81 (p < 0.001), r = 0.76 (p < 0.001) and r = 0.78 (p < 0.001) with ICCs of 0.80 (95% CI 0.77–0.82), 0.75 (95% CI 0.72–0.78) and 0.65 (95% CI 0.61–0.69), respectively. Figure 5 shows scatter plots comparing strain measures between 3D-CMR and 3D-STE.

### 3D CMR-based strain versus 2D CMR-based strain

Further validation of 3D-CMR PS was sought against the current reference standards of 2D tagged cine analysis and 2D feature tracking cine analysis. In the former analysis, strong agreement was identified between 3D-CMR maximum PS and tagged MRI analysis of longitudinal, circumferential and radial strain showing respective correlations of r = −0.81, r = −0.85, and r = 0.81, respectively (p < 0.0001 for all). Strong agreement was also identified between 3D-CMR minimum PS and tagged MRI analysis of longitudinal, circumferential and radial strain showing respective correlations of r = 0.81, r = 0.87, and r = −0.76 (p < 0.0001 for all). Pearson correlation coefficients for longitudinal, circumferential and radial strain between the two techniques were as follows: r = 0.87, r = 0.88, and r = 0.75, respectively (p < 0.0001 for all). Intra-class correlation coefficients (ICC) were 0.89, 95% (CI 0.88–0.90), 0.84 (95%CI 0.82–0.86) and 0.88 (95% CI 0.86–0.89), respectively (p < 0.0001 for all).

Strong agreement was identified between 3D-CMR maximum PS and 2D cine feature tracking measures of longitudinal, circumferential and radial strain showing respective correlations of r = −0.83, r = −0.87, and r = 0.84, respectively (p < 0.0001 for all). Strong agreement was also identified between 3D-CMR minimum PS and 2D cine feature tracking analysis of longitudinal, circumferential and radial strain showing respective correlations of r = 0.85, r = 0.88, and r = −0.79 (p < 0.0001 for all). Pearson correlation coefficients for longitudinal, circumferential and radial strain between the two techniques were as follows: r = 0.91, r = 0.93, and r = 0.77, respectively (p < 0.0001 for all). Corresponding ICC values were 0.85 (95% CI 0.83–0.87), 0.85 (95% CI 0.83–0.87), and 0.88 (95% CI 0.86–0.89), respectively (p < 0.0001 for all).

Between like measures, comparisons were made using strain measures in geometry-dependent directions from both 3D and corresponding 2D techniques. Bland–Altman analysis showed mean error of measurement between 3D-CMR and 2D tagged-MR based strain to be low at 5.4 ± 13.8% for radial strain, 1.5 ± 3.0% for circumferential strain, and 0.1 ± 2.8% for longitudinal strain. The corresponding mean error versus 2D feature-tracking was −0.5 ± 13.6% for radial strain, 2.9 ± 3.5% for circumferential strain, and 1.4 ± 3.0% for longitudinal strain (Figure C1, Supplementary material).

### Internal validation versus global systolic function

Internal (within modality) validation versus non-strain based measures of systolic performance was sought through correlation of 3D-CMR PS and LV EF, as measured via CMR from endocardial contours using an independent, FDA-approved software (cvi42, Circle Cardiovascular Inc). As shown in Table 4, significant Pearson correlation coefficients were found between LVEF and both maximum and minimum PS, similar to those achieved for conventional strain measures in geometry-dependent directions. As expected, measures derived from the endocardial surfaces showed stronger association with LVEF (also derived from the endocardial surface).

**Table 4:** Correlation coefficients for peak systolic left ventricular 3-dimensional strain values versus left ventricular ejection fraction measured by cardiovascular magnetic resonance imaging

Parameter	r-value
Conventional strain	
Longitudinal	
Endocardial	−0.70 (p < 0.001)
Epicardial	−0.57 (p < 0.001)
Transmural	−0.65 (p < 0.001)
Circumferential	
Endocardial	−0.82 (p < 0.001)
Epicardial	−0.49 (p = 0.005)
Transmural	−0.71 (p < 0.001)
Radial	
Transmural	0.60 (p < 0.001)
Principal strain	
Minimum	
Endocardial	−0.75 (p < 0.001)
Epicardial	−0.40 (p = 0.024)
Transmural	−0.62 (p < 0.001)
Maximum	
Transmural	0.53 (p < 0.001)

Data are expressed as Pearson correlation coefficient (r) (p-value)

### Reproducibility analyses

Intra-observer reproducibility for CMR-based 3D LV strain calculations are presented in Table 5 for strain measures in both principal and geometry-dependent directions. Excellent reproducibility (p < 0.001 for all directions) was seen with ICC values between 0.83 and 0.98. PS analysis provided higher reproducibility (0.92–0.98) than analyses in geometry-dependent directions. Inter-observer variability was similarly robust (ICC range 0.83–0.97) and similarly was found to be strongest for PS-based measurements. Corresponding Bland–Altman plots are provided in Supplementary material. The biases identified for intra-observer variability were −0.96 ± 1.07, 2.2 ± 17, and −0.99 ± 0.93% for longitudinal, radial, and circumferential strain respectively. Corresponding biases for maximum and minimum principal strain were −0.75 ± 0.88 and −0.2 ± 6.4%, respectively. Biases for inter-observer variability were 0.5 ± 2.0, −0.9 ± 13.0, 0.52 ± 1.94% for longitudinal, radial, and circumferential strain, respectively. Corresponding biases for maximum and minimum principal strain were 0.7 ± 7.6 and 0.52 ± 1.6%, respectively.

**Table 5:** Intra-observer and inter-observer assessments for peak systolic left ventricular 3-dimensional strain measures

Parameter	Intra-observer ICC (95% CI)	Inter-observer ICC (95% CI)
Conventional strain		
Longitudinal		
Endocardial	0.88 (0.63–0.96)	0.83 (0.50–0.95)
Epicardial	0.89 (0.66–0.97)	0.87 (0.60–0.06)
Transmural	0.91 (0.71–0.97)	0.89 (0.66–0.97)
Circumferential		
Endocardial	0.92 (0.74–0.98)	0.89 (0.66–0.97)
Epicardial	0.91 (0.71–0.97)	0.89 (0.65–0.96)
Transmural	0.91 (0.71–0.97)	0.89 (0.66–0.97)
Radial		
Transmural	0.83 (0.50–0.95)	0.87 (0.60–0.96)
Principal strain		
Minimum		
Endocardial	0.93 (0.77–0.98)	0.91 (0.71–0.97)
Epicardial	0.92 (0.74–0.98)	0.88 (0.63–0.96)
Transmural	0.93 (0.77–0.98)	0.92 (0.74–0.98)
Maximum		
Transmural	0.98 (0.93–0.99)	0.97 (0.90–0.99)

Data are presented as the Inter-class correlation coefficient (ICC) (95% confidence interval, CI). P < 0.001 for all directions. *CI* confidence interval, *ICC* intra-class correlation, *p* p-value

## Discussion

This study validates a novel approach to performing 3D myocardial strain analysis from routine 2D cine CMR images for the description of principal strain. This technique was shown to be clinically feasible, provide high correlation to conventional strain measures using both 2D CMR and 3D STE techniques, and showed high reproducibility. Most importantly, this work supports the application of 3D PS as a geometry-independent measure of tissue deformation discarding assumptions of chamber architecture.

The expanded clinical use of CMR offers a unique platform to develop and validate 3D data models. Its superior soft-tissue contrast and high temporal resolution make it ideal for the development of high quality, cohort-specific models aimed at improved diagnostic accuracy and the prediction of adverse outcomes. The incremental capacity of CMR to provide for the validation of underlying tissue pathology through the use of Late Gadolinium Enhancement (LGE) and tissue mapping techniques further establishes its unique value in this role. While the concept of 3D myocardial strain computation from CMR datasets has been previously explored [4, 21–31], both using tagged cine images [22, 23] and 3D displacement encoding with simulated echoes (DENSE) [21, 24–27], resultant analyses have mimicked those provided by 2D strain. Further, these techniques require the use of additional pulse sequences not routinely applied in clinical practice. One such study, published by Ahmed et al., described the 3D reconstruction of 2D tagged cine images in an effort to establish a 4D deformation field [30], the latter aim similar to our currently described approach. While effective, this study required the incremental acquisition of LAX and SAX tagged cine images.

To our knowledge, only one other study has described the use of routine, non-tagged cine images to develop a 4D displacement field for the deformation of a mesh-based LV model, this performed in a small number of dogs using gradient-echo cine imaging [28, 29]. Using a shape-based tracking approach strain measures were calculated and found to be highly consistent with those obtained by the in-vivo tracking of image-opaque markers surgically implanted at the epicardial, endocardial and mid-myocardial levels of the mid LV [29]. This pre-clinical study did not explore measures of deformation in geometry-independent directions, such as PS, and therefore did not fully exploit the value of this approach.

PS analysis provides a composite measure of tissue deformation in the dominant direction of local tissue deformation, and may therefore more accurately reflect activation of regional tissue myofibers [3]. The removal of constraints applied through pre-determined geometry-dependent directions of deformation may therefore provide a more accurate and reproducible measure of local myofiber disease. Evidence supporting this claim remains preliminary, and is based on several pre-clinical studies that have consistently identified PS to reflect deformation in the direction of local myofibers [3, 32–34]. Recognizing expanded validation is required, such a tool would address findings by Fonseca et al. identifying heterogeneous alterations in LV kinematics occurring following the inception of tissue pathology [31], further supporting disease may not conform to pre-defined axes of deformation. This said, we do anticipate conventional strain parameters (i.e. longitudinal strain) to retain their strong clinical role given well established clinical adoption and expanding value, particularly among specific patient populations [35].

Zhong et al. described that maximum PS (dominant direction of tissue thickening) approximated radial deformation while minimum PS (dominant direction of tissue shortening) approximated its perpendicular plane [26]. This evidence was further corroborated by Pedrizzetti, et al. using 3D STE images acquired in 41 healthy subjects [3]. Using the same commercial 3D-STE software employed in our current study, they described peak endocardial PS to be concordant with myocardial fiber orientation, as derived by diffusion tensor imaging (DTI) MRI [3]. Incremental to these reports, we provide sentinel evidence of clinical feasibility in patients with suspected cardiovascular disease from routine cine CMR, and demonstrate strong correlations between 3D PS and contemporary measures of 2D strain.

The correlation coefficients of 3D PS to conventional strain values that we obtained from STE ranged from moderate to very strong (0.4–1.0) versus more consistently high correlations (0.8–1.0) for within-modality comparisons. This is an anticipated finding given cross-modality variations in acquisition technique, soft tissue definition, and use of independent feature tracking software techniques. Of note, the best correlations for 3D PS were, in general, obtained versus longitudinal strain, reflecting a more consistent relationship of this conventional strain marker to net (i.e. principal) deformations, irrespective of the modality evaluated. This aligns with expanding observations for longitudinal strain being the most valuable clinical marker (of conventional strain parameters) for the assessment of myocardial disease.

In this study, we performed extensive validation of 3D PS versus conventional measures of strain in geometry-dependent directions as measured by 2D tagged cine imaging, 2D non-tagged cine imaging, and 3D STE each measured by technique appropriate, FDA-approved commercial software. Consistently we identified strong, significant correlations between 3D PS and measures of longitudinal, circumferential and radial strain. These findings establish 3D PS to be a simplified yet comparable marker of tissue deformation versus contemporary methodology.

## Limitations

Several limitations to our study must be recognized. First, 3D STE strain may be considered an imperfect clinical reference due to modest temporal resolution and the single surface tracking methodology employed by the available analysis software. The latter introduces the potential for under-representation of transmural strain given that the spatial location of the epicardial surface must be estimated. Second, the mean interval between CMR and 3D STE was 26 ± 15 days. This introduces a potential for alterations in loading conditions between these examinations. However, given that this is anticipated to reduce agreement between techniques, our observed findings are robust and should be considered a modest estimate of agreement.

Several technical developments surrounding improved analysis automation are feasible but were not tested in this study. For example, manual rigid registration was employed to adjust for changes in cardiac position on repeat breath-holds (as necessary) and manual LAX chamber tracing was used to initialize a mesh model. While not significantly impacting clinical feasibility, methodologies for automated motion correction and chamber segmentation have since been developed and will be introduced in future studies.

The present study was designed to demonstrate clinical feasibility and provide comparisons of 3D PS versus other conventional strain techniques in a relevant referral population. Accordingly, reference values among healthy volunteers were not obtained. Such reference values are being prospectively sought in a healthy cohort study and will be published separately.

It is important to recognize that the temporal resolution of routine cine CMR imaging remains inferior to that achievable by 2D echocardiography. Therefore, while our described approach offers unique opportunities to explore 3D measures of cardiac deformation through using high quality CMR images, frequency-sensitive measures of deformation (i.e. myocardial activation timing) may remain best studied by high temporal resolution echocardiographic techniques.

Finally, as a feasibility and validation study, this technique was investigated in consecutively recruited clinical patients referred for CMR imaging for any indication (other than congenital heart disease). Accordingly, while a wealth of regional and temporally-encoded data is available from our described technique, exploration of these measures in a mixed referral cohort was not appropriate. Current efforts are therefore aimed towards exploring these unique markers within large, well-defined disease cohorts in addition to establishing age and gender-specific reference atlas datasets.

## Conclusion

3D principal strain analysis from routine 2D cine CMR imaging is clinical feasible, highly reproducible, and shows strong correlations with conventional (geometry-dependent) measures of strain. This approach does not require additional sequences and can be applied retrospectively to historic CMR datasets. As such, this technique provides unique opportunities to apply a single definition of strain (principal directions) that may accurately describe mechanical alterations related to local myocardial fiber disease. Studies investigating the applicability of this strain analysis in various disease cohorts are underway.

### Electronic supplementary material

Below is the link to the electronic supplementary material.

Supplementary material 1 **Online Supplemental Video**. Video illustrating 3D maximum principal strain of the left ventricle (both endocardial and epicardial surfaces) throughout the cardiac cycle in a patient with normal left ventricular size and function. Red color represents higher strain values. (MOV 2382 KB)

Supplementary material 2 (DOCX 960 KB)

## Appendix 1: strain analysis (detailed mathematics)

### Left ventricular mesh model reconstruction

The $x$, $y$ coordinates of each manually drawn 2D LAX contour are converted to patient space space ($x$, $y$ and $z$ coordinates) through the use of accompanying DICOM metadata. Each LAX contour (2, 3 and 4 chamber) is considered to initiate at the mitral annulus and terminate at the apex, establishing 2 independent walls for each view. A set of ${N}_{\text{Level}}$ equidistant nodes are obtained from resampling of each of the contours, where each $k$-th level represents a cross section of the left ventricle, with $k=1:{N}_\text{Level}$.

The 6 nodes corresponding to the $k$-th level of both the endocardial and epicardial contours are then fitted in a best-fit plane (BP) with ${x}_{BP}$and ${y}_{BP}$ coordinates estimated via an ellipse equation, whereas the ${z}_{BP}$ coordinate is fit quadratically. On this final shape ${N}_{Slice} = 24$ equidistant nodes are established, and re-converted to patient space. Given the $j$-th node on the $i$-th level a 4-node EL_Epi_ element (13) is defined on the epicardial surface from the nodes with $ij$-th, $i(j+1)$-th, $(i+1)j$-th and $(i+1)(j+1)$-th indices. A corresponding 4-node endocardial EL_Endo_ element is defined with the same indices as EL_Epi_. Finally, a solid 8-node hexahedral EL_Transmural_ element (14,32) is established with vertices defined as the nodes of EL_Epi_ and the nodes of EL_Endo_. Using three element definitions allows being able to estimate strain at the subendocardial and epicardial surfaces, while at the same time taking advantage of a uniform deformation gradient hexahedron to compute transmural strain considering interactions between the two surfaces.

### 2D tracking and mesh deformation

At every time step $n$, the in-plane velocity is calculated for each pixel in an image by comparing phase $n$ and $n+1$, through a feature-based optical tracking algorithm (15–17). Velocities are computed for every pixel in every imaging plane at every phase. In practice, this is done by looking at the pixel specific image brightness and how it changes in time and in space: thus, the algorithm computes the pixel-specific in-plane velocity components ${v}_{x}$ and ${v}_{y}$ at which a pixel in phase $n$ should move in order for its appearance (defined by the brightness of the pixel and its surrounding pixels) to be similar at phase $n+1$. Accordingly, the pixel-specific vector with components $({v}_{x},{v}_{y})$ defines completely the in-plane pixel-specific velocity. The algorithm computes in-plane velocities taking into account that neighboring pixels will have similar velocities and the velocity field varies smoothly.

The in-plane velocity computed for each pixel is then converted into its 3-dimensional representation (in patient space) according to the accompanied DICOM header coordinates. This in turn is used to inform displacement of the LV mesh, where the velocity of each node in patient-space ($x$, $y$and $z$) directions are weighted by the velocities of the closest pixel of each imaging plane with inverse proportionality to their distance (between node and pixel). Every weight also accounts for inverse proportionality to the dot product between the normal of the cine imaging plane and the patient-space direction in which the velocity is investigated. Therefore, while the distance weight is equal for all 3 patient-space directions, the dot product (i.e. angulation) weight component changes for each of the 3 interrogated directions. The computed velocity between time steps *n* and *n* + 1 is expressed in terms of spatial units ($mm$) per time step, thus allowing us to consider velocity as numerically equal to the incremental displacement that the material point undergoes between time steps. Because the 4D velocity field that displaces the nodes of the mesh is reconstructed using the in-plane velocity information obtained in multiple planes (SAX and LAX planes), the out-of-plane resolution of each cine SSFP 2D image does not limit the resolution the obtained 4D velocity field, since it is compensated by the finer resolution provided by images in planes orthogonal orthogonal to each other.

With regards to the element formulations, current and initial geometries are defined based on the initial ${X}_{a}$ and current ${x}_{a}$ nodal positions, as:

$${\mathbf{X}}=\mathop \sum \limits_{a=1}^N {X_a}{N_a}({\mathbf{\xi }})$$

$${\mathbf{x}}=\mathop \sum \limits_{a=1}^N {x_a}{N_a}({\mathbf{\xi }})$$

where $N$ is the number of nodes defining the element, ${N}_{a}\left(\mathbf{\xi }\right)$ are the appropriate shape functions.

The spatial derivative of the mapping $\mathbf{x}=\varphi \left(\mathbf{X}\right)$ relating the two configurations yields the deformation gradient tensor $\mathbf{F}$:

$${\mathbf{F}}=\mathop \sum \limits_{a=1}^N {x_a} \otimes {\nabla _0}{N_a}$$

### Strain evaluation

For every EL_Transmural_ and corresponding EL_Endo_ and El_Epi_ elements, deformation gradient $\mathbf{F}$ is computed as described by Hallquist [13]. The element’s Green–Lagrange strain tensor is therefore obtained as:

$${\mathbf{E}}=\frac{1}{2}\left( {{{\mathbf{F}}^T}{\mathbf{F}} - {\mathbf{I}}} \right)$$

where $\mathbf{I}$ is the $3\times$3 identity matrix. After defining the nodal radial, circumferential and longitudinal directions as ${\mathbf{u}}_{RR}$, ${\mathbf{u}}_{CC}$ and ${\mathbf{u}}_{LL}$ respectively, the Green–Lagrange strain in the $KK$-th direction ${\mathbf{u}}_{KK}$ is defined as:

$${E_{KK}}={\mathbf{E}}:({{\mathbf{u}}_{KK}} \wedge {{\mathbf{u}}_{KK}})$$

where ${\mathbf{u}}_{KK}$ is the vector describing the K-th direction. From every 4-node element EL_endo_, subendocardial circumferential, longitudinal and minimum principal strain are obtained. From every 4-node element EL_Epi_, subepicardial circumferential, longitudinal and minimum principal strain are obtained. From every 8-node EL_Transmural_ element, transmural minimum and maximum principal, radial, circumferential and longitudinal strain are computed. Principal strain is computed as eigenvalues of $\mathbf{E}$.

## Abbreviations

2D: 2-dimensional
3D: 3-dimensional
4D: 4-dimensional
CI: Confidence interval
CMR: Cardiovascular magnetic resonance imaging
DICOM: Digital imaging and communications in medicine
EF: Ejection fraction
ICC: Intra-class correlation coefficient
LAX: Long-axis
LV: Left ventricle (ventricular)
PS: Principal strain
SAX: Short-axis
SSFP: Steady-state free precession
