# Supplementary material for: Clinical feasibility and validation of 3D principal strain analysis from cine MRI: comparison to 2D strain by MRI and 3D speckle tracking echocardiography
Source: Int J Cardiovasc Imaging. 2017 Jul 6;33(12):1979–92. doi: 10.1007/s10554-017-1199-7 (PMC5698377; doi:10.1007/s10554-017-1199-7)
Supplement: Supplementary file 2 — Supplementary material 2 (DOCX 960 KB) [file 10554_2017_1199_MOESM2_ESM.docx]

**APPENDIX B – Supplementary Figures**

**
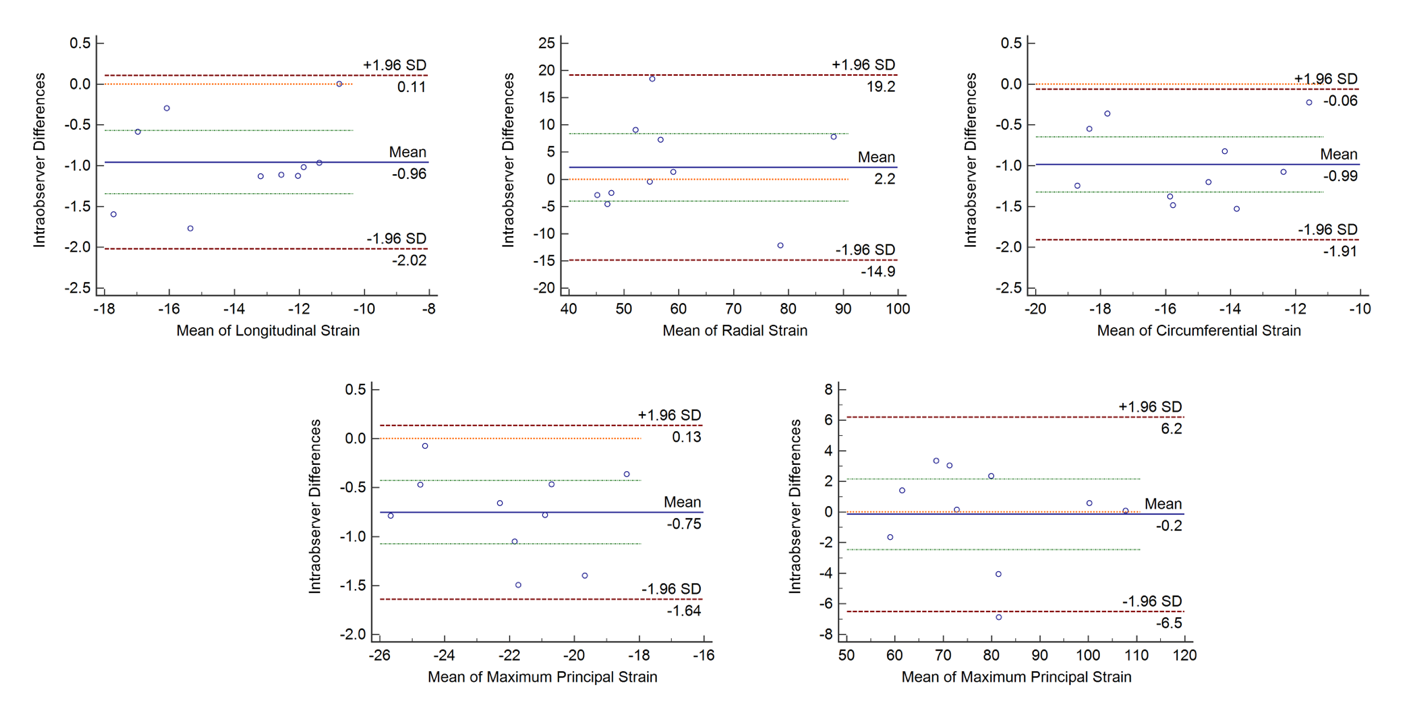
**

**Figure B1:** Bland-Altman plots for intra-observer variability of transmural measures of strain.

**
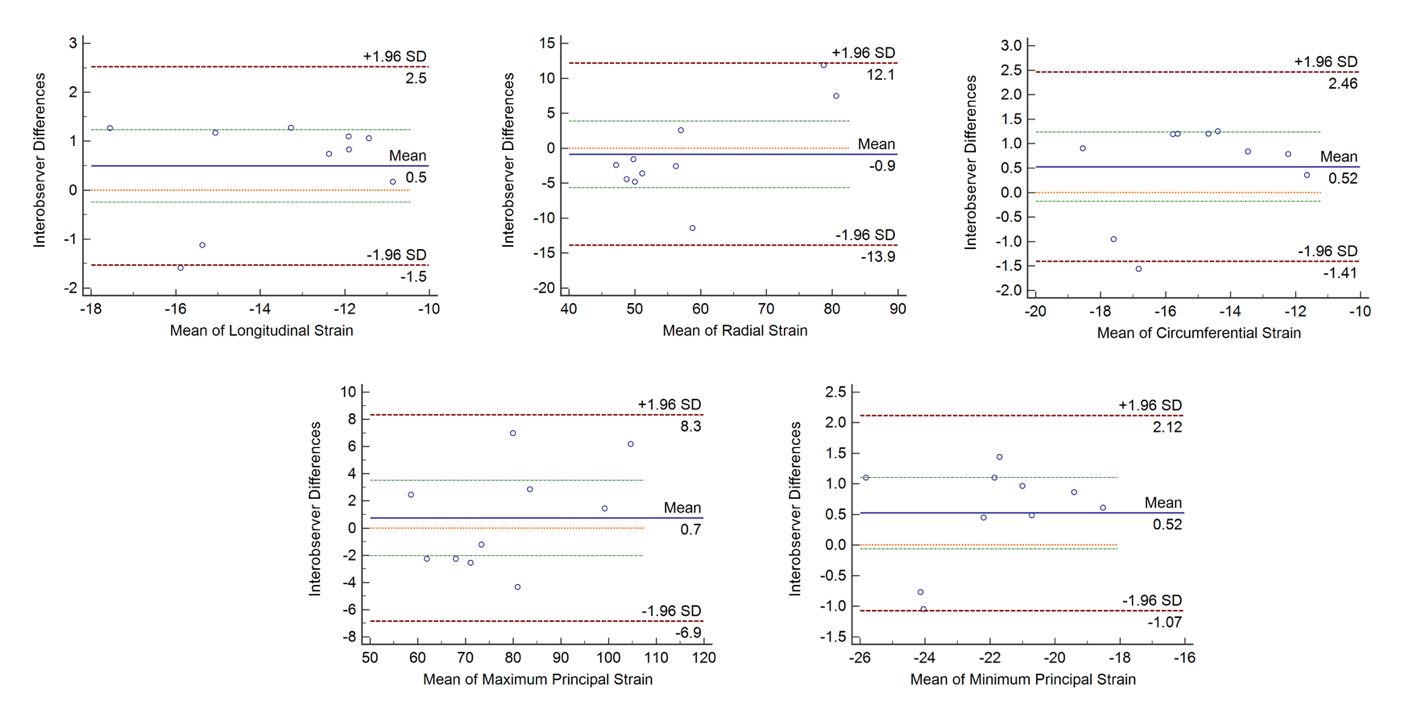
**

**Figure B2:** Bland-Altman plots for inter-observer variability of transmural measures of strain.

**APPENDIX C – Comparison between MR-Based 3D and 2D strain**

**Figure C1:** Bland-Altman analyses comparing in radial, circumferential and longitudinal directions 3D feature-tracking based strain to 2D tagged-MR based and 2D feature-tracking based strain analysis. FT, Feature Tracking.
